# Matrix remodeling-associated protein 5 as a novel biomarker for predicting disease activity and endoscopic response to infliximab in Crohn's disease

**DOI:** 10.1016/j.jtauto.2025.100300

**Published:** 2025-07-07

**Authors:** Daopo Lin, Jiayue Xu, Mengqian Ye, Luyan Fang, Tianhao Xia, Wenyu Tong, Gokuljayanth Jayaseelan Ranichandra, Yifan Bao, Bo Zheng, Yi Jiang, Lianpin Wu, Dingyuan Hu

**Affiliations:** a Department of Gastroenterology, The Second Affiliated Hospital and Yuying Children's Hospital of Wenzhou Medical University, Wenzhou, 325000, China; b Department of Neurology, The Second Affiliated Hospital and Yuying Children's Hospital of Wenzhou Medical University, Wenzhou, 325000, China; c The Second School of Medicine, Wenzhou Medical University, Wenzhou, 325000, China; d Department of Cardiology, The Second Affiliated Hospital and Yuying Children's Hospital of Wenzhou Medical University, Wenzhou, 325000, China; e Wenzhou Key Laboratory of Precision General Practice and Health Management, Wenzhou, 325000, China

**Keywords:** Crohn's disease, Biomarker, Proteomics, MXRA5

## Abstract

The primary objective of treating Crohn's disease (CD) is to achieve and sustain endoscopic remission. However, repeated endoscopic examination leads to decreased patient compliance and procedural risks. Non-invasive biomarkers for endoscopic activity of CD are thus promising in clinical use. This study compared proteomic profiles between inflammatory and non-inflammatory intestinal tissues on 10 active CD patients through liquid chromatography–tandem mass spectrometry, and identified 384 differentially expressed proteins. Four candidate secretory proteins (MXRA5, AZU/HBP, CRYAB, DEFA3) were validated via ELISA in serum from 74 CD patients (43 active CD and 31 in remission). Serum MXRA5 levels were notably increased in CD patients in remission compared to active cases (*P* < 0.001) and showed an inverse correlation with SES-CD scores (r = −0.33, *P* < 0.05). ROC analysis demonstrated MXRA5's utility in distinguishing endoscopic activity of patients with CD (AUC = 0.80), which was improved when combined with CRP (AUC = 0.89). Besides, higher baseline serum MXRA5 levels predicted better endoscopic response to infliximab (IFX). In conclusion, our study indicates that MXRA5 might serve as a new potential serum biomarker for CD activity and IFX response prediction. Further prospective and muli-center studies are needed to validate its predictive performance.

## 1 Introduction

Crohn's disease (CD) is a chronic, relapsing gastrointestinal condition with increasing prevalence and incidence in both industrialized and developing countries [1]. The activity of CD can be evaluated through clinical symptoms, endoscopic observations, radiologic assessments, or microscopic examination [2]. Presently, the primary objective of therapeutic interventions is to achieve and sustain endoscopic remission. In clinical practice, assessing disease response through ileocolonoscopy and imaging requires a 3–6 months interval to allow for repair cycles, after which healing is re-evaluated using endoscopy or imaging. Therapy is then adjusted based on these findings, with subsequent evaluations repeated [2]. Despite of being the most effective current method, the routine use of repeated colonoscopy in clinical practice or trials may be limited by patient discomfort, procedural risks, anesthesia needs, and high costs. Consequently, there is a pressing need for novel biomarkers that can accurately reflect disease activity as observed through endoscopic analyses.

Despite substantial advancements in the discovery of multi-omic biomarkers, none have yet been integrated into routine clinical practice. Biomarkers like C-reactive protein (CRP) are clinically useful for evaluating disease susceptibility, activity, and behavior [3]. CRP is non-specific and may be elevated in various inflammatory conditions, making it challenging to distinguish CD activity from other causes. Furthermore, CRP shows a limited correlation with endoscopic disease activity, with a sensitivity of only 49 % for identifying endoscopically active CD [3]. Fecal calprotectin (FC) is currently recognized as the most reliable and accurate protein biomarker for diagnosing inflammatory bowel disease (IBD) [4]. Recent randomized trial data suggest that early therapeutic interventions guided by FC biomarkers can improve disease outcomes in CD [5]. Despite its use in clinical care, fecal testing has notable limitations [6], highlighting the need for blood-based alternatives.

The protein domain is arguably the most universally impacted in disease processes, responses, and recovery, rendering proteomics particularly promising for biomarker discovery [7]. Over the past decade, significant progress has been made in refining proteomic technologies to identify novel drug targets and molecular signatures linked to clinically significant diseases, specific disease subsets, or different therapeutic responses [8]. Recent proteomic studies and validation efforts have significantly increased the range of potential diagnostic and prognostic biomarkers for CD. For example, Zhou et al. [9] identified differential expression of proteins, including prohibitin, calreticulin, apolipoprotein A-I, intelectin-1, protein disulfide isomerase, and glutathione S-transferase Pi, in inflamed versus normal intestinal mucosa. Similarly, Han et al. [10] observed elevated levels of bone marrow proteoglycan, L-plastin, and proteasome activator subunit 1 in active CD. Moriggi M et al. [11] investigated the proteomic profiles of inflamed and non-inflamed mucosal areas in 30 CD patients. The study revealed that inflamed mucosal areas in CD patients showed increased expression of collagen type VI alpha 1 chain and vimentin. Despite these findings, the biomarkers were predominantly investigated in small population cohorts, necessitating further validation before they can be utilized clinically.

This study aimed to use comparative proteomics to identify the proteomic signature distinguishing inflammatory from non-inflammatory colonic tissue in ten CD patients, establishing baseline markers for active CD. Based on the proteomic results from intestinal tissues, we identified innovative intestinal secretory proteins and validated them in the peripheral blood of a second cohort of CD patients, thereby further investigating their potential clinical relevance. These analyses are anticipated to enhance our understanding of CD pathogenesis and facilitate the discovery of novel therapeutic targets.

## 2 Materials and methods

### 2.1 Study population and design

This study examined two patient groups admitted to the Gastroenterology Department at the Second Affiliated Hospital of Wenzhou Medical University from September 2020 to September 2021. All participants fulfilled the diagnostic criteria specified in the 3rd European evidence-based consensus for CD diagnosis and treatment [12]. We collected demographic, clinical, and colonoscopic data from each subject's medical records. Patient characteristics documented at inclusion comprised age at diagnosis, gender, smoking history, Montreal classification, treatment history, endoscopic disease activity assessed via the Simplified Endoscopic Activity Score for Crohn's Disease (SES-CD), and endoscopic pathological findings. The SES-CD, a standard tool in China for assessing CD activity [13], categorizes scores as follows: less than 3 indicates remission, 3–6 mild disease, 7–15 moderate disease, and over 15 severe disease [14]. Patients in remission scored below 3, while active disease was indicated by scores of 3 or higher. The hospital's ethics committee approved the study (Ethical Application Reference 2024-K-289-01).

Proteomic and histological analyses were conducted on intestinal mucosal tissue samples from 10 patients with active CD in cohort A. Samples were collected from the ulcer edge in the ileum, with paired control biopsies from adjacent macroscopically normal ileal mucosa. These biopsies were collected in the endoscopy suite following a standardized protocol. Subsequently, the tissues were flash-frozen and preserved in liquid nitrogen.

A total of 74 serum samples from 68 CD patients were collected for analysis via Enzyme-Linked Immunosorbent Assay (ELISA), including 6 samples from the same patients at different time points. In cohort B, 43 patients with active CD were treated with infliximab (IFX) following a standardized intravenous regimen: 5 mg/kg at weeks 0, 2, and 6, with subsequent maintenance every 8 weeks. Therapeutic response was defined as a 50 % or greater reduction in SES-CD at week 24 after initial IFX treatment, indicating an endoscopic response. Non-response was characterized by either no improvement or deterioration in endoscopic findings or disease symptoms.

### 2.2 Liquid chromatography–tandem mass spectrometry (LC-MS/MS) analysis

Frozen samples were analyzed using a proteomics approach based on LC-MS/MS. Proteins were extracted from the samples, and their concentrations were quantified using the Micro BCA kit (Thermo Scientific Pierce). Following protein extraction, samples were reduced with 10 mM dithiothreitol (DTT) for 10 min at 95 °C and subsequently alkylated with 40 mM iodoacetamide for 20 min at 24 °C in the dark. The samples were then precipitated with acetone for 2 h at −20 °C. Following precipitation, samples were centrifuged at 14,000 g for 15 min at 4 °C, and the supernatant was removed with care. The pellets were dissolved in a solution of 50 mM ammonium bicarbonate with 5 % acetonitrile. Samples were digested overnight at 37 °C using trypsin at a protein-to-trypsin mass ratio of 70:1. The digested peptides were desalted using Oasis HLB cartridges (1 cc/30 mg, Waters).

Peptides were loaded onto a C18 trap column (Acclaim PepMap RSLC, 75 μm × 15 cm, nanoViper C18, 2 μm particles, 100 Å pore size, Thermo Scientific, San José, CA) with a flow rate of 300 nL/min. A nonlinear gradient was applied using solvent A (0.1 % formic acid) and solvent B (80 % acetonitrile with 0.1 % formic acid). The gradient was programmed to transition from 8 % to 28 % solvent B over the first 70 min, increase to 35 % solvent B over the next 10 min, and finally rise to 100 % solvent B over 6 min.

Protein digests were analyzed with an Orbitrap Fusion Lumos mass spectrometer and an Easy-nLC 1200 pump (Thermo Scientific, San José, CA) using a top 10 data-dependent acquisition (DDA) strategy. Full MS scans were performed using the Orbitrap mass analyzer over an *m/z* range of 350–1500, with a resolution of 120,000, an AGC target of 4.0 × 10^5^, and a maximum injection time of 50 ms. The top ten peaks with a charge state of 2 or higher were fragmented in the higher-energy collisional dissociation (HCD) cell using a normalized collision energy of 30 %. Tandem mass spectra were acquired using the Orbitrap mass analyzer at a resolution of 30,000, with an AGC target of 5.0 × 10^4^ and a maximum injection time of 54 ms. The ion selection threshold was established at 4.2 × 10^4^, with a dynamic exclusion duration of 20 s.

### 2.3 ELISA

A total of 74 serum samples were obtained from 68 patients diagnosed with CD, forming an independent cohort separate from those subjected to LC-MS/MS analysis. Blood samples were drawn from the ulnar vein after fasting and centrifuged at 2000×*g* for 20 min. The serum was subsequently stored at −80 °C. ELISA on four selected proteins [Matrix remodeling-associated protein 5 (MXRA5), Azurocidin/Heparin-Binding Protein (AZU/HBP), Crystallin Alpha B (CRYAB), and Neutrophil defensin 3 (DEFA3)] were conducted in accordance with the manufacturer's protocols (Boyun Biotech, China).

### 2.4 Bioinformatics and statistics

The mass spectrum data and retention times for each run were systematically collected by the platform for subsequent analysis. The data were processed using Protein Discoverer 2.4, with the Uniprot Homo sapiens database (UP000005640) serving as the reference database. A decoy database, comprising reversed versions of all protein sequences, was incorporated to monitor the false discovery rate (FDR). Peptide identification was conducted with precursor mass tolerance set to 20 ppm and fragment mass tolerance at 0.5 Da. Oxidation and carbamidomethylation modifications were considered, and up to two missed cleavage sites were permitted. Proteins were identified by detecting a minimum of two high-confidence peptides, maintaining an FDR below 1 %.

Perseus software [15] was employed for statistical analysis. Protein intensities were normalized to their median values and subsequently subjected to Log2 transformation to facilitate further statistical evaluation. Protein intensities between the two groups were compared using a paired *t*-test. Hierarchical clustering and principal component analysis were conducted to identify significant intergroup differences. The bioinformatics analysis of interaction networks among differentially expressed proteins was conducted using STRING [16].

The Shapiro-Wilk W test was employed to evaluate the normality of continuous variables. Continuous variables were presented as mean ± standard error for normal distributions and as median (interquartile range) for non-normal distributions. Continuous variables were compared using Student's t-test or the Mann-Whitney *U* test. Categorical variables were expressed as percentages or rates and analyzed with the chi-square or Fisher's exact test. Statistical correlations were examined through Spearman's rank correlation analysis. Receiver Operating Characteristic (ROC) and binary logistic regression analyses were conducted using SPSS, with statistical significance set at *P* < 0.05. Variables with a variance inflation factor below 10, indicating no collinearity, were chosen for multivariate analysis if they showed statistical significance (*P* < 0.2) in univariate logistic regression. The strength of associations was reported as odds ratios (OR) with 95 % confidence intervals (CI). Statistical significance was defined as a two-sided *P*-value of less than 0.05.

## 3 Results

### 3.1 Descriptive patient characteristics

Our research methodology is illustrated in Supplementary Fig. 1, which delineates the two cohorts we assembled. Cohort A included 10 patients with active CD who underwent proteomics analyses, with their specific characteristics detailed in Table 1. Cohort B consisted of 74 patients, including 43 with active CD and 31 with remitting CD, used to validate the predictive potential of specific proteins. Table 2 provides detailed characteristics of the study population.

**Table 1:** Baseline characteristics of active Crohn's disease patients in the proteomics cohort.

ID	Age, years	Gender	Smoking history	SES-CD	Classification	Treatment history
CD_1	26	Male	Never	30	A2L3B2p	IFX
CD_2	32	Male	Never	16	A2L3B2	NA
CD_3	18	Male	Never	11	A2L2B2	NA
CD_4	43	Male	Never	5	A3L3B1	5-ASA
CD_5	19	Male	Never	4	A2L3B3	5-ASA, AZA
CD_6	28	Female	Never	11	A2L3B2	NA
CD_7	54	Female	Never	15	A3L2B1	NA
CD_8	18	Male	Never	20	A2L2B1	AZA, IFX, glucocorticoid
CD_9	34	Female	Never	10	A2L3B2	5-ASA, IFX
CD_10	32	Male	Never	13	A2L3+L4B3	5-ASA, AZA, glucocorticoid

**Abbreviations:** CD, Crohn's disease; SES-CD, Simple Endoscopic Score for Crohn's Disease; Classification, Montreal classification (phenotype codes: A = age at diagnosis, L = disease location, B = disease behavior); Treatment history: IFX, Infliximab (anti-TNF therapy); 5-ASA, 5-aminosalicylic acid; AZA, Azathioprine; NA: No prior therapy.

**Table 2:** Baseline characteristics of Crohn's disease patients stratified by disease activity status.

Variable	Overall, N = 74^a^	Disease status	Active, N = 43 (58 %)^a^	*p*-value^b^
		Remission, N = 31 (42 %)^a^		
Age at diagnosis, years	22.50 [19.00, 31.00]	25.00 [20.00, 34.00]	22.00 [18.50, 28.50]	0.150
Gender				0.628
Male	49 (66.22 %)	22 (70.97 %)	27 (62.79 %)	
Female	25 (33.78 %)	9 (29.03 %)	16 (37.21 %)	
Smoking history				0.325
No	60 (81.08 %)	23 (74.19 %)	37 (86.05 %)	
Yes	14 (18.92 %)	8 (25.81 %)	6 (13.95 %)	
Perianal lesions				>0.999
No	29 (39.19 %)	12 (38.71 %)	17 (39.53 %)	
Yes	45 (60.81 %)	19 (61.29 %)	26 (60.47 %)	
Disease behavior				0.764
B1	49 (66.22 %)	21 (67.74 %)	28 (65.12 %)	
B2	17 (22.97 %)	6 (19.35 %)	11 (25.58 %)	
B3	8 (10.81 %)	4 (12.90 %)	4 (9.30 %)	
Disease extent				0.418
L1±L4	15 (20.27 %)	4 (12.90 %)	11 (25.58 %)	
L2±L4	9 (12.16 %)	4 (12.90 %)	5 (11.63 %)	
L3±L4	50 (67.57 %)	23 (74.19 %)	27 (62.79 %)	
Prior surgical history				0.644
No	69 (93.24 %)	28 (90.32 %)	41 (95.35 %)	
Yes	5 (6.76 %)	3 (9.68 %)	2 (4.65 %)	
Previous use of biologics				<0.001
No	36 (48.65 %)	4 (12.90 %)	32 (74.42 %)	
Yes	38 (51.35 %)	27 (87.10 %)	11 (25.58 %)	
Disease duration, years	1.00 [0.50, 3.00]	1.20 [0.90, 2.40]	0.60 [0.20, 3.00]	0.023
CRP, mg/L	4.38 [0.80, 23.48]	0.79 [0.32, 3.07]	14.30 [3.34, 46.52]	<0.001
Albumin, g/L	42.24 (3.93)	43.85 (3.03)	41.08 (4.13)	0.003
Platelets, × 10^9^/L	262.50 [220.00, 350.25]	225.00 [181.50, 269.50]	302.00 [252.00, 392.50]	<0.001
WBC, × 10^9^/L	6.50 [5.41, 8.27]	6.47 [5.29, 7.97]	6.52 [5.63, 8.55]	0.290
ESR, mm/h	14.00 [4.00, 31.00]	4.00 [2.00, 9.00]	23.00 [11.00, 43.50]	<0.001
Hemoglobin, g/L	136.27 (22.35)	146.19 (16.89)	129.12 (23.22)	<0.001

**^Abbreviations:^**^CRP, C−reactive protein; WBC, White Blood Cell Count; ESR, Erythrocyte Sedimentation Rate; Disease extent was defined as being ileal (L1), colonic (L2), ileocolonic (L3) and upper gastrointestinal (L4); Disease behavior was stratified as non-penetrating, non-stricturing (B1), stricturing (B2), or penetrating disease (B3).^

a Median [IQR]; n (%); Mean (SD).

b Wilcoxon rank sum test; Pearson's Chi-squared test; Fisher's Exact Test for Count Data with simulated p-value (based on 2000 replicates); Fisher's exact test

### 3.2 Candidate prognostic proteins for CD

**Fig. 1:**
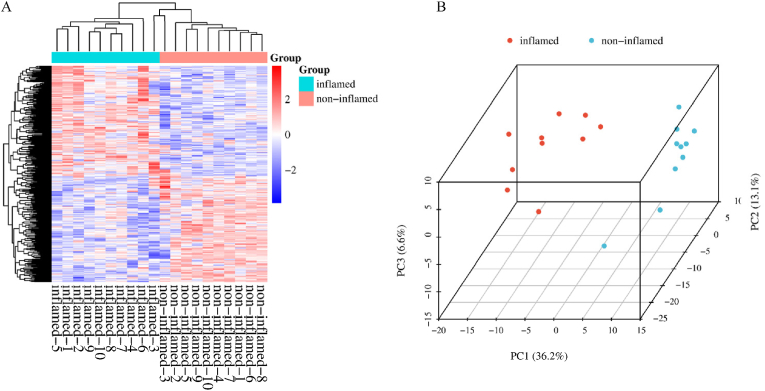
Heat map and principal component analysis of differentially expressed proteins in inflammatory and non-inflammatory intestinal tissues of Crohn's d**isease. A:** Heat map visualized two-way unsupervised hierarchical clustering of 384 differentially expressed proteins in inflammatory and non-inflammatory intestinal tissues from 10 patients with Crohn's disease (*P* < 0.05, fold change ≥2 or ≤0.5). **B:** Global principal component analysis of protein profiles in paired inflammatory (red colored) and non-inflammatory (blue-colored) intestinal tissues from 10 patients with Crohn's disease.

**Fig. 2:**
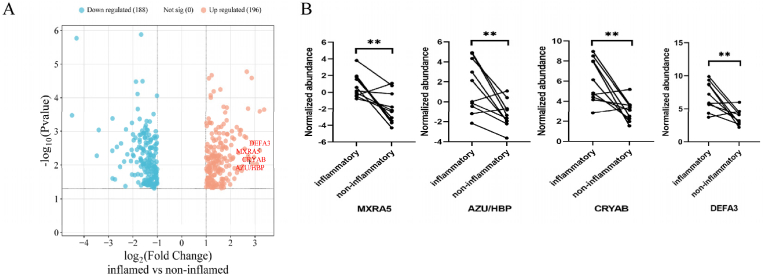
Differential protein expressions in inflammatory and non-inflammatory intestinal tissues of Crohn's d**isease. A:** Volcano plot illustrating the differential expressed proteins between inflamed and non-inflamed regions of intestinal tissue in Crohn's disease. Proteins with significant upregulation (red) and downregulation (blue) are highlighted, with the x-axis representing the log2 fold change and the y-axis showing the -log10 p-value. **B:** Expression levels of four selected differentially expressed proteins (MXRA5, AZU1/HBP, CRYAB, and DEFA3) in paired inflamed versus non-inflamed tissue.

Initially, we conducted a comparative analysis of the protein composition between inflammatory and non-inflammatory intestinal tissues from patients with active CD. Our study quantitatively identified a total of 5252 proteins, of which 384 exhibited differential expression between the inflammatory and non-inflammatory regions. These proteins satisfied the criteria of an inflammatory/non-inflammatory fold change of ≥2 or ≤0.5, with a significance level of *P* < 0.05. Supplementary Table 1 details the top 10 upregulated and top 10 downregulated proteins in inflammatory versus non-inflammatory sites in CD patients. A heat map (Fig. 1A) visualizes the results of two-way unsupervised hierarchical clustering applied to the 384 differentially expressed proteins. The clustering of the 20 tissue samples corresponded well with their clinical classification. Principal component analysis (PCA) confirmed the distinct stratification of samples from inflammatory (red) and non-inflammatory (blue) intestinal tissues based on differentially expressed proteins (Fig. 1B). Within this set, 196 proteins were overexpressed, while 188 proteins were under-expressed in patients with inflammatory regions compared to non-inflammatory groups (Fig. 2A). We employed the STRING database to investigate the functional and physical interactions among the 384 differentially expressed proteins (Supplementary Fig. 2). We categorized the unique metabolites using Kyoto Encyclopedia of Genes and Genomes (KEGG) pathway enrichment to assess their potential functions. The analysis identified that the primary pathways affected between the two stages of CD, with an FDR threshold <0.05, include complement and coagulation, phagosome, prion disease, metabolic pathways, Staphylococcus aureus infection, IL-17 signaling, systemic lupus erythematosus, PPAR signaling, proximal tubule bicarbonate reclamation, and Alzheimer's disease.

Through a comprehensive review of the literature, we have identified relatively novel secretory proteins among the 384 differential expressed proteins. These proteins, specifically MXRA5, AZU/HBP, CRYAB, and DEFA3, were all upregulated in inflammatory lesions compared to non-inflammatory regions of CD, as illustrated in Fig. 2B.

### 3.3 Serum ELISA validation of biomarker candidates

**Fig. 3:**
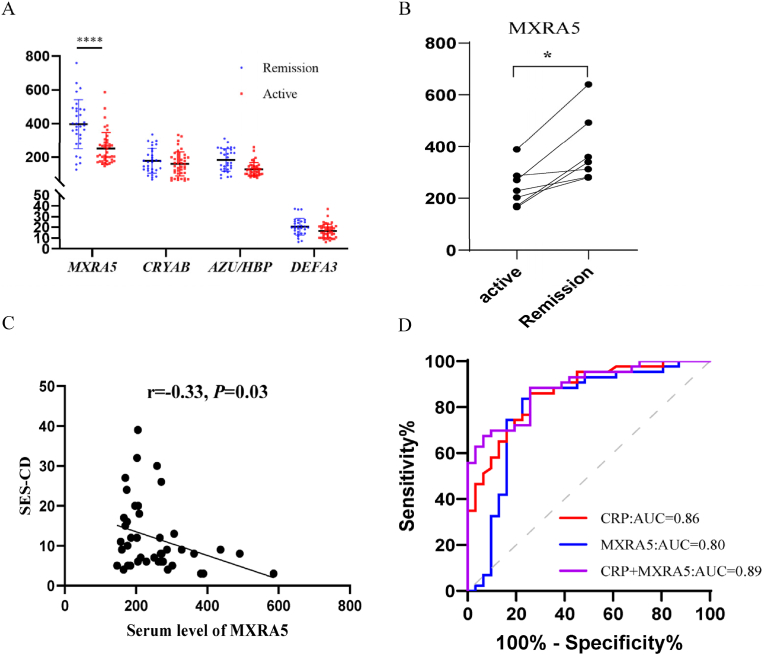
Expression levels of selected proteins in peripheral blood of CD patients and their predictive pot**ential. A:** Expression levels of four proteins (MXRA5, AZU1/HBP, CRYAB, and DEFA3) in peripheral blood during active and remission phases of CD. The mean relative abundance of each protein was presented, with comparisons between active and remission phases. **B:** Longitudinal analysis of protein expression levels in a subset of CD patients with paired samples (active vs. remission phases). The changes in expression levels of MXRA5, AZU1/HBP, CRYAB, and DEFA3 within the same individuals over time were shown. **C:** Inverse relationship between serum MXRA5 levels and SES-CD (Simple Endoscopic Score for CD) scores in patients with CD. **D:** Receiver operating characteristic (ROC) curves evaluating the predictive performance of MXRA5, CRP, and their combination in distinguishing active CD from remission CD. The area under the curve (AUC) values were provided for MXRA5, CRP, and the combined model. CD: Crohn's disease.

An ELISA analysis was subsequently conducted to validate four candidate biomarkers. Fig. 3A demonstrates that MXRA5 expression in peripheral blood is significantly higher in CD patients in remission than active cases (396.60 ± 26.23 vs. 252.13 ± 14.45 ng/mL, *P* < 0.001). No statistically significant differences were found between the two groups for AZU/HBP, CRYAB, and DEFA3. Notably, among the 74 CD serum specimens analyzed in this study, samples from both active and remitting phases were simultaneously collected from six patients. Analysis of MXRA5 protein expression in the peripheral blood of the six patients showed increased levels during the remitting phase compared to the active phase (386.61 ± 50.37 vs. 244.72 ± 29.73 ng/mL, *P* < 0.05) (Fig. 3B). Spearman's rank correlation analysis was conducted to investigate the association between serum MXRA5 expression levels and SES-CD scores. Fig. 3C illustrates a negative correlation between serum MXRA5 expression levels and SES-CD scores in active CD patients (r = −0.33, *P* < 0.05).

Based on these findings, we are poised to further investigate the clinical relevance of the MXRA5 protein in CD. To assess the potential of MXRA5 in differentiating between active and remission phases in CD patients, we employed the ROC curve analysis, which yielded an area under the curve (AUC) value of 0.8 (Fig. 3D). MXRA5 levels above 309 ng/ml were identified as indicative of mucosal healing, with a sensitivity of 83.7 % and a specificity of 77.4 %. The positive and negative predictive values were 72.7 % and 86.8 %, respectively. Given that serum CRP levels are well-established as a biomarker for distinguishing active and remission phases in CD, we also analyzed CRP levels within this cohort, obtaining an AUC value of 0.86. Moreover, the combined analysis of blood MXRA5 levels and serum CRP enhanced the predictive accuracy, resulting in an increased AUC value of 0.89 (Fig. 3D).

### 3.4 Serum MXRA5 level can predict the endoscopic response to IFX

In this study, all 43 patients with active CD underwent treatment with IFX, with 26 patients (60 %) demonstrating an endoscopic response at week 24. Table 3 presents the baseline characteristics of the patient cohort. At week 0, CD patients who were responsive to IFX had significantly higher serum MXRA5 levels than those who did not respond (270.50 [207.00, 305.75] vs. 184.00 [170.00, 206.00] ng/mL, *P* < 0.001). The two groups showed no significant differences in age, gender, smoking history, disease extent, disease behavior, or SES-CD scores.

**Table 3:** Baseline characteristics of Crohn's disease patients stratified by endoscopic response to infliximab at week 24.

Variable	Overall, N = 43^a^	IFX treatment		statistics	*p*-value^b^
		No response, N = 17 (40 %)^a^	Response, N = 26 (60 %)^a^		
Age at diagnosis, years	22.00 [18.50, 28.50]	22.00 [19.00, 34.00]	22.00 [18.00, 26.75]	247.50	0.517
Gender					0.910
Male	27 (62.79 %)	10 (58.82 %)	17 (65.38 %)		
Female	16 (37.21 %)	7 (41.18 %)	9 (34.62 %)		
Smoking history					0.376
No	37 (86.05 %)	16 (94.12 %)	21 (80.77 %)		
Yes	6 (13.95 %)	1 (5.88 %)	5 (19.23 %)		
Perianal lesions					0.115
No	17 (39.53 %)	4 (23.53 %)	13 (50.00 %)		
Yes	26 (60.47 %)	13 (76.47 %)	13 (50.00 %)		
Disease behavior					0.098
B1	28 (65.12 %)	8 (47.06 %)	20 (76.92 %)		
B2	11 (25.58 %)	7 (41.18 %)	4 (15.38 %)		
B3	4 (9.30 %)	2 (11.76 %)	2 (7.69 %)		
Disease extent					0.364
L1±L4	9 (20.93 %)	4 (23.53 %)	5 (19.23 %)		
L2±L4	7 (16.28 %)	1 (5.88 %)	6 (23.08 %)		
L3±L4	27 (62.79 %)	12 (70.59 %)	15 (57.69 %)		
Prior surgical history					0.511
No	41 (95.35 %)	17 (100.00 %)	24 (92.31 %)		
Yes	2 (4.65 %)	0 (0.00 %)	2 (7.69 %)		
Previous use of biologics				0.00	>0.999
No	31 (72.09 %)	12 (70.59 %)	19 (73.08 %)		
Yes	12 (27.91 %)	5 (29.41 %)	7 (26.92 %)		
Disease duration, years	0.80 [0.20, 3.00]	0.60 [0.20, 1.00]	1.00 [0.27, 3.00]	173.00	0.235
CRP, mg/L	14.30 [3.34, 46.52]	20.40 [12.00, 38.90]	6.86 [3.11, 64.29]	258.00	0.365
Albumin, g/L	41.09 (4.13)	39.72 (3.47)	41.99 (4.33)	−1.90	0.065
Platelets, × 10^9^/L	302.00 [252.00, 392.50]	317.00 [262.00, 388.00]	299.00 [239.25, 415.75]	237.00	0.700
WBC, × 10^9^/L	6.42 [5.63, 8.40]	6.95 [5.68, 8.29]	6.31 [5.53, 8.33]	240.00	0.646
MXRA5, ng/ml	213.00 [180.50, 281.50]	184.00 [170.00, 206.00]	270.50 [207.00, 305.75]	77.50	<0.001
ESR, mm/h	25.00 [11.00, 43.50]	28.00 [18.00, 39.00]	22.00 [7.50, 44.00]	233.50	0.765
Hemoglobin, g/L	128.72 (23.23)	127.65 (26.25)	129.42 (21.55)	−0.23	0.818
SES-CD	9.00 [6.00, 15.50]	11.00 [7.00, 16.00]	8.00 [6.00, 12.75]	246.00	0.542

**^Abbreviations:^**^IFX, Infliximab;CRP, C−reactive protein; WBC, White Blood Cell Count; SES−CD, Simple Endoscopic Score for Crohn’ s Disease; ESR, Erythrocyte Sedimentation Rate; MXRA5, Matrix Remodeling-associated 5; Disease extent was defined as being ileal (L1), colonic (L2), ileocolonic (L3) and upper gastrointestinal (L4); Disease behavior was stratified as non-penetrating, non-stricturing (B1), stricturing (B2), or penetrating disease (B3).^

a Median [IQR]; n (%); Mean (SD).

b Wilcoxon rank sum test; Fisher's exact test; Fisher's Exact Test for Count Data with simulated p-value (based on 2000 replicates); Pearson's Chi-squared test; Welch Two Sample *t*-test

Univariable logistic regression analysis revealed significant associations between disease behavior (OR = 0.267, 95 % CI 0.071–0.997) and serum MXRA5 levels (OR = 1.019, 95 % CI 1.005–1.033) with the endoscopic response to IFX at week 24 in CD patients (Table 4). No significant associations were found between the endoscopic response to IFX in CD patients and perianal lesions (OR = 0.308, 95 % CI 0.079–1.198), disease extent (OR = 0.208, 95 % CI 0.023–1.912), or albumin levels (OR = 1.157, 95 % CI 0.981–1.363).

**Table 4:** Univariate and Multivariate Logistic Regression Analysis for Risk Factors associated with Endoscopic Response to Infliximab at Week 24 in Crohn's Disease Patients.

Variable	UniVariate *OR* (95 % *CI*)	*P* value	MultiVariate *OR* (95 % *CI*)	*P* value
Age	0.985 (0.918–1.057)	0.677		
Gender	1.322 (0.375–4.658)	0.664		
Disease extent	0.208 (0.023–1.912)	0.165	0.232 (0.016–3.317)	0.282
Disease behavior	0.267 (0.071–0.997)	0.049	4.098 (0.645–26.042)	0.135
Perianal lesions	0.308 (0.079–1.198)	0.089	0.169 (0.029–0.971)	0.046
Smoking history	0.263 (0.028–2.474)	0.243		
Disease duration	1.074 (0.779–1.481)	0.661		
Previous use of biologics	1.131 (0.291–4.390)	0.859		
MXRA5	1.019 (1.005–1.033)	0.009	1.018 (1.003–1.034)	0.020
CRP	1.003 (0.981–1.026)	0.761		
WBC	0.971 (0.784–1.203)	0.789		
Hemoglobin	1.003 (0.977–1.031)	0.804		
Platelets	1.000 (0.994–1.005)	0.905		
Albumin	1.157 (0.981–1.363)	0.082	1.111 (0.907–1.360)	0.310
ESR	1.002 (0.978–1.027)	0.868		
SES-CD	1.002 (0.933–1.077)	0.953		

Abbreviations: CRP, C-reactive protein; WBC, White Blood Cell Count; SES-CD, Simple Endoscopic Score for Crohn's Disease; ESR, Erythrocyte Sedimentation Rate; MXRA5, Matrix Remodeling-associated 5.

Gender was transformed as categorical variables where female = 1, male = 0.

Disease extent was transformed as categorical variables where L1/L3 = 1, L2 = 0.

Disease behavior was transformed as categorical variables where B2/B3 = 1, B1 = 0.

Perianal lesions, smoking history and previous use of biologics were all transformed as categorical variables where “Yes” = 1, “No” = 0.

A multivariate logistic regression analysis was conducted to address the influence of confounding variables. Variables with a *P*-value <0.2 were incorporated into the multivariable analysis. After adjusting for potential confounders, multivariate analysis identified serum MXRA5 levels (OR = 1.018, 95 % CI 1.003–1.034) and perianal lesions (OR = 0.169, 95 % CI 0.029–0.971) as predictors of endoscopic response at week 24 in Crohn's Disease patients receiving IFX therapy.

## 4 Discussion

This study presents a preliminary proteomic analysis comparing inflammatory and non-inflammatory intestinal tissue samples from patients with active CD. We identified 384 differentially expressed proteins. KEGG pathway analysis indicated that these proteins were predominantly enriched in the complement and coagulation cascades, phagosome, and prion disease pathways. The interaction between the coagulation and complement cascades in CD is implicated not only in the acute inflammatory response but may also influence the chronic progression and recurrence of the disease [17]. Moreover, research by Choung et al. [18] demonstrated that the coagulation and complement cascades are significantly upregulated in preclinical serum samples of patients with complications at diagnosis, compared to those with non-complicated CD, suggesting an association between these cascades and the complicated CD phenotype at diagnosis. Phagosomes are essential for initiating the immune response in CD by identifying and engulfing pathogens [19]. Phagosome function may be compromised during the disease's active phase, leading to decreased pathogen clearance and increased inflammation [20]. Activation and inhibition of these pathways play an important role in disease activity in CD, providing potential targets for future therapies.

Serum biomarker detection, typically achieved through minimally invasive blood sampling, is well tolerated by patients and provides insights into systemic pathological and physiological alterations. This method allows for dynamic monitoring of disease progression and therapeutic efficacy, facilitating timely adjustments to treatment plans. To identify serum markers that accurately reflect intestinal inflammation in CD, four novel secretory proteins (MXRA5, CRYAB, AZU/HBP, and DEFA3) from a pool of the 384 differentially expressed proteins were selected and further validated by peripheral blood ELISA.

In this study, the expression of MXRA5 was significantly elevated in inflammatory intestinal tissues of CD compared to the non-inflammatory tissues. An ELISA study revealed significantly higher serum MXRA5 expression in CD patients in remission compared to active cases. Although the function of MXRA5 in CD has not been investigated, its anti-inflammatory and anti-fibrotic properties in response to fibrogenic cytokine transforming growth factor-β1 and pro-inflammatory stimuli have been proposed in kidney [21]. We assume that MXRA5 overexpression is initiated in inflamed intestine of CD and play a favorable role in the healing process of CD. This hypothesis may both explain the overexpression of MXRA5 in inflamed tissues as compared to non-inflamed tissues, and the overexpression of MXRA5 in CD patients with better mucosal healing (in remission). Nevertheless, further mechanisms study of the protective role of MXRA5 should be investigated. MXRA5 is a secretory glycoprotein with seven leucine-rich repeats and 12 immunoglobulin-like C2-type domains, essential for cell adhesion and extracellular matrix (ECM) remodeling [22]. MXRA5 shows substantial expression alterations in multiple tumors, such as colorectal cancer, and is strongly linked to tumor progression, invasion, and prognosis. MXRA5 potentially aids tumor progression by enhancing cell proliferation, migration, and epithelial-mesenchymal transition, making it a viable therapeutic target for various tumors [22,23]. ECM remodeling is significantly intensified during the active phase of CD [24]. Matrix metalloproteinases (MMPs) are upregulated in CD, facilitating ECM degradation and remodeling. This process promotes the infiltration of inflammatory cells, resulting in tissue destruction [25]. Adhesion molecules play a crucial role in maintaining the integrity and function of intestinal epithelial cells. In the active phase of CD, changes in adhesion molecule expression can weaken intestinal epithelial cell tight junctions, disrupting the intestinal barrier function [26]. Consequently, the involvement of MXRA5 in ECM remodeling and cell adhesion suggests its potential as a significant biomarker and therapeutic target in the context of CD.

We utilized the ROC curve to assess MXRA5's effectiveness in differentiating between active and remitting phases in CD patients, resulting in an AUC of 0.8. CRP is an acute-phase reactant that exhibits a rapid increase during inflammatory responses and serves as a crucial marker for assessing inflammation severity [3]. In CD patients, elevated CRP levels are typically associated with active intestinal inflammation. However, CRP levels can also rise in response to other inflammatory diseases, infections, trauma, and various conditions. Factors such as diet, medication, and stress can influence CRP levels, and its dynamic fluctuations may not fully capture the true activity of intestinal inflammation. Therefore, it is imperative to integrate additional indicators for a comprehensive evaluation. Our study determined that the AUC value for predicting CD activity using CRP alone is 0.86, demonstrating a high level of accuracy, yet indicating potential for enhancement. Upon integrating MXRA5 with CRP, the AUC value increased to 0.89. The result shows that the combined detection can more accurately predict the activity of CD.

IFX, a monoclonal antibody targeting TNF-α, is extensively utilized in the management of CD and has demonstrated efficacy in inducing clinical remission among affected patients. Recent research indicates that certain factors, including HLA-DQA1∗05 gene polymorphisms, elevated CRP levels, reduced albumin concentrations, increased disease activity index scores, and the presence of perianal lesions, may be associated with a diminished clinical response to IFX [[27], [28], [29]]. The study found that patients who did not respond to IFX had significantly lower pre-treatment serum levels of MXRA5 compared to those who did respond. Individuals with reduced serum MXRA5 levels exhibited a higher likelihood of experiencing a loss of response to IFX, as corroborated by both univariable and multivariate logistic regression analyses. Multivariate analysis revealed that perianal lesions independently predict a reduced endoscopic response to IFX. The study indicates that serum MXRA5 levels could be a new biomarker for predicting IFX's therapeutic efficacy in CD treatment.

CRYAB, a small heat shock protein, serves as a molecular chaperone essential for protein folding, stabilization, and transport within cells [30]. Recent research has indicated that elevated expression of CRYAB is associated with increased invasiveness and metastasis in several solid tumor types [31]. Conversely, during enterovirus infection, the degradation and phosphorylation of CRYAB promote viral replication and contribute to viral pathogenesis [32]. Our study has found elevated CRYAB expression in the intestinal inflammatory tissues of CD patients compared to non-inflammatory tissues. However, the result failed to be confirmed in serum level between patients with active CD and those CD in remission. Xu et al. [30] reported a significant reduction of CRYAB in the inflamed mucosa of patients with IBD and in mice with dextran sulfate sodium induced colitis. This reduction was negatively correlated with the levels of TNF-α and IL-6, respectively. Enforced CRYAB expression suppresses proinflammatory cytokines (e.g., TNF-α, IL-6, IL-1β, IL-8) by inhibiting IKK complex formation, while its absence enhances proinflammatory responses. The variation in results could be attributed to sample selection differences, our study aimed at paired comparison of inflammatory and non-inflammatory tissue from patients with active CD, while Xu et al. compared the intestinal tissues of patients with active CD and CD in remission.

AZU/HBP is a prevalent antimicrobial protein found in neutrophils, characterized by its broad-spectrum antimicrobial properties, which enable it to effectively eradicate a variety of bacteria, fungi, and viruses [33]. DEFA3, a cationic peptide from the defensin family, exhibits antimicrobial properties against bacteria, fungi, and viruses, and also modulates immune cell activity and inflammatory responses [34]. Currently, there are no studies examining the relationship between AZU/HBP and DEFA3 in the context of active CD. Although our research indicated elevated AZU/HBP and DEFA3 levels in inflammatory intestinal tissues of active CD compared to non-inflammatory tissues, their serum expression showed no difference between patients with active CD and those CD in remission. Further studies might be helpful to illustrate the potential role of AZU/HBP and DEFA3 in context of CD.

However, this study has several limitations. First, the assessment of MXRA5 dynamics was limited to two timepoints (active disease vs. remission), a more comprehensive longitudinal analysis with multiple sampling points during therapy induction and maintenance would better characterize its translational utility for monitoring treatment response. Second, although paired baseline and post-therapy serum MXRA5 data were available for a small subset of IFX responders (N = 6), the sample size was underpowered to definitively confirm MXRA5 restoration kinetics. Future studies should include dense longitudinal serum sampling across therapeutic stages, and validation of MXRA5 restoration patterns in larger IFX-treated cohorts to fully establish its clinical utility.

In conclusion, our findings indicate that MXRA5 emerges as a promising biomarker candidate for assessing disease activity and forecasting therapeutic outcomes in patients with CD. The integration of MXRA5 with CRP improves the capacity to differentiate active and inactive CD patients. Furthermore, serum MXRA5 concentrations, in conjunction with the presence of perianal lesions, may act as valuable predictors of endoscopic response to IFX, thereby offering potential clinical utility in the development of personalized treatment strategies for CD.

## CRediT authorship contribution statement

**Daopo Lin:** Writing – original draft, Methodology, Investigation, Data curation, Conceptualization. **Jiayue Xu:** Writing – original draft, Methodology, Investigation, Data curation, Conceptualization. **Mengqian Ye:** Writing – original draft, Methodology, Investigation, Data curation, Conceptualization. **Luyan Fang:** Visualization, Validation, Formal analysis. **Tianhao Xia:** Visualization, Validation, Formal analysis. **Wenyu Tong:** Visualization, Validation, Formal analysis. **Gokuljayanth Jayaseelan Ranichandra:** Software, Resources, Data curation. **Yifan Bao:** Software, Resources, Data curation. **Bo Zheng:** Supervision, Project administration. **Yi Jiang:** Supervision, Project administration. **Lianpin Wu:** Writing – review & editing, Supervision, Funding acquisition. **Dingyuan Hu:** Writing – review & editing, Supervision, Project administration, Conceptualization.

## Funding

This work was supported by grants from 10.13039/501100004731Zhejiang Provincial Natural Science Foundation [Grant number: LY23H030004].

## Declaration of competing interest

The authors declare that they have no known competing financial interests or personal relationships that could have appeared to influence the work reported in this paper.

## Data Availability

Data will be made available on request.
