# Supplementary material for: Matrix remodeling-associated protein 5 as a novel biomarker for predicting disease activity and endoscopic response to infliximab in Crohn's disease
Source: J Transl Autoimmun. 2025 Jul 7;11:100300. doi: 10.1016/j.jtauto.2025.100300 (PMC12275958; doi:10.1016/j.jtauto.2025.100300)
Supplement: Multimedia component 1 [file mmc1.docx]

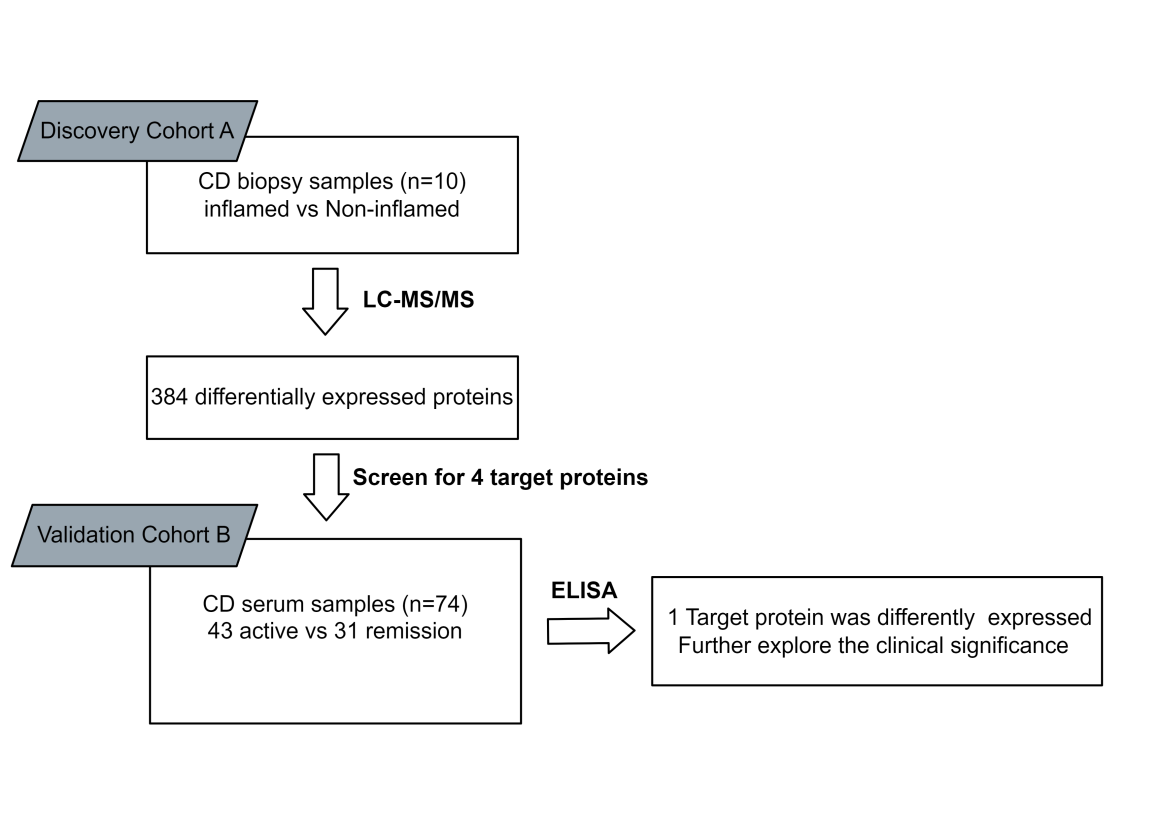


**Supplementary Fig. 1. Study flow chart.** CD, Crohn’s disease


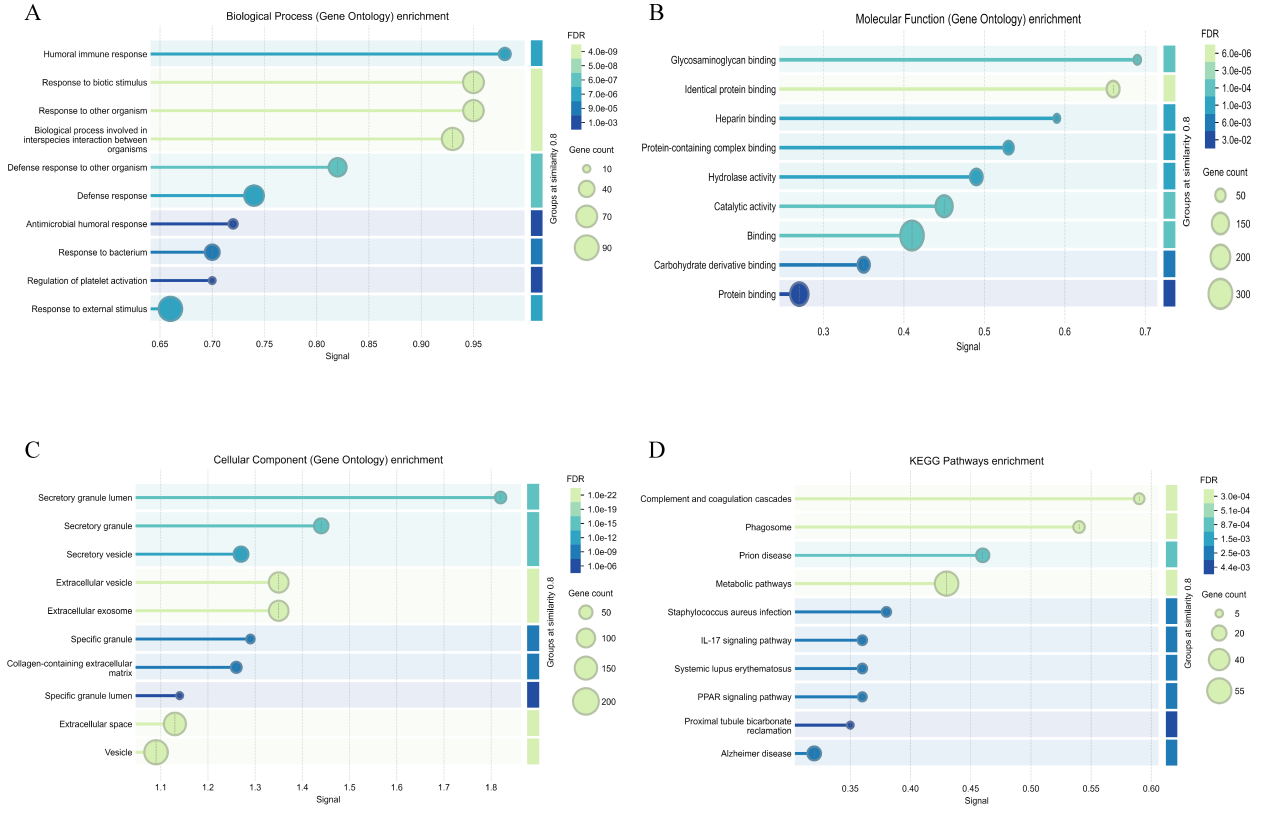


**Supplementary Fig. 2. Functional annotation of differentially expressed proteins in inflammatory and non-inflammatory intestinal tissues of Crohn's disease using the STRING database.** **A:** Biological Process enrichment analysis; **B:** Molecular Function enrichment analysis; **C:** Cellular Component enrichment analysis; **D:** KEGG pathway enrichment analysis. Each panel represents the functional annotation of proteins identified through proteomic comparison, providing insights into the biological relevance of these proteins in the context of Crohn’s disease.

| **Supplementary Table 1.**  Proteomic analysis of Crohn’s disease lesions: Top 10 upregulated and top 10 downregulated proteins in inflammatory vs. non-inflammatory tissues. | | | | |
| --- | --- | --- | --- | --- |
| Entry | Gene | Description | Fold change | *P* value |
| Q6PD62 | CTR9 | RNA polymerase-associated protein CTR9 homolog | 10.38 | <0.001 |
| P59666 | DEFA3 | Neutrophil defensin 3 | 9.27 | 0.003 |
| Q04446 | GBE1 | 1,4-alpha-glucan-branching enzyme | 9.20 | <0.001 |
| P02057 | HBB2 | Hemoglobin subunit beta-1/2 | 8.49 | 0.009 |
| P02511 | CRYAB | Alpha-crystallin B chain | 7.85 | 0.006 |
| P42768 | WAS | Wiskott-Aldrich syndrome protein | 7.52 | 0.009 |
| Q13136 | LOC105199525 | Liprin-alpha-1 | 7.49 | 0.002 |
| P55058 | PLTP | Phospholipid transfer protein | 7.29 | <0.001 |
| P20160 | AZU/HBP | Azurocidin/Heparin-Binding Protein | 6.95 | 0.007 |
| Q9NR99 | MXRA5 | Matrix-remodeling-associated protein 5 | 6.65 | 0.005 |
| Q9BVJ7 | DUSP23 | Dual specificity protein phosphatase 23 | 0.17 | 0.011 |
| Q5VT66 | MTARC1 | Mitochondrial amidoxime-reducing component 1 | 0.16 | 0.003 |
| Q562R1 | ACTBL2 | Beta-actin-like protein 2 | 0.15 | 0.027 |
| P02088 | HBB-B1 | Hemoglobin subunit beta-1 | 0.14 | <0.001 |
| Q9Y3R5 | DOPEY2 | Protein dopey-2 | 0.14 | 0.004 |
| P11836 | MS4A1 | B-lymphocyte antigen CD20 | 0.14 | 0.025 |
| O75312 | ZPR1 | Zinc finger protein ZPR1 | 0.10 | 0.001 |
| P03969 | FGF2 | Fibroblast growth factor 2 | 0.09 | 0.005 |
| P35443 | THBS4 | Thrombospondin-4 | 0.05 | <0.001 |
| O95218 | ZRANB2 | Zinc finger Ran-binding domain-containing protein 2 | 0.04 | <0.001 |
